# Outcomes from a multimodal, at‐scale community‐based HIV counselling and testing programme in twelve high HIV burden districts in South Africa

**DOI:** 10.1002/jia2.25678

**Published:** 2021-03-11

**Authors:** Andrew Medina‐Marino, Joseph Daniels, Dana Bezuidenhout, Remco Peters, Thato Farirai, Jean Slabbert, Geoffrey Guloba, Suzanne Johnson, Linda‐Gail Bekker, Nkhensani Nkhwashu

**Affiliations:** ^1^ Research Unit Foundation for Professional Development East London South Africa; ^2^ The Desmond Tutu HIV Centre University of Cape Town Cape Town South Africa; ^3^ Perelman School of Medicine University of Pennsylvania Philadelphia USA; ^4^ Department of Psychiatry and Human Behaviors Charles R. Drew University of Medicine and Science Los Angeles CA USA; ^5^ Community‐based HIV Counselling and Testing Program Foundation for Professional Development Pretoria South Africa

**Keywords:** HIV testing services, HIV testing uptake, community‐based HIV counselling and testing, sex disparities, adolescent girls and young women, South Africa

## Abstract

**Introduction:**

Facility‐based HIV testing services (HTS) have been less acceptable and accessible by adolescents, men and key populations in South Africa. Community‐based HIV counselling and testing (CBCT) modalities, including mobile unit and home‐based testing, have been proposed to decrease barriers to HIV testing uptake. CBCT modalities and approaches may be differentially acceptable to men and women based on age. Implementation of multimodal CBCT services may improve HIV testing rates among adolescents and men, and support the roll‐out of prevention services.

**Methods:**

A cross‐sectional analysis was conducted using aggregate, routine programmatic data collected from 1 October 2015 through 31 March 2017 from a multimodal, at‐scale CBCT programme implemented in 12 high‐burden districts throughout South Africa. Data collection tools were aligned to reporting standards for the National Department of Health and donor requirements. HIV testing rates (i.e. number of tests performed per 100,000 population using South African census data) and testing proportions by modality were stratified by sex, age groups and heath districts. Descriptive statistics were performed using STATA 13.0.

**Results:**

Overall, 944,487 tests were performed during the 1.5‐year testing period reported. More tests were conducted among females than males (53.6% vs. 46.4%). Overall, 8206 tests per 100,000 population (95% CI: 8190.2 to 8221.9) were performed; female‐to‐male (F:M) testing ratio was 1.11. Testing rates were highest among young women age 20 to 24 years (16,328.4; 95% CI: 16,237.9 to 16,419.1) and adolescent girls aged 15 to 19 years (12,817.0; 95% CI: 12,727.9 to 12,906.6). Home‐based testing accounted for 61.3% of HIV tests, followed by near‐home mobile unit testing (30.2%) and workplace mobile unit testing (4.7%). More women received HTS via home‐based testing (F:M ratio = 1.29), whereas more men accessed work‐place mobile testing (M:F ratio = 1.35). No sex differential was observed among those accessing near‐home mobile testing (F:M ratio = 0.98).

**Conclusions:**

Concurrent implementation of multiple, targeted CBCT modalities can reduce sex disparities in HIV testing in South Africa. Given the acceptability and accessibility of these CBCT services to adolescent girls and young women, evident from their high testing rates, leveraging community‐based services delivery platforms to increase access to HIV prevention services, including pre‐exposure prophylaxis (PrEP), should be considered.

## 1 INTRODUCTION

South Africa has the largest HIV epidemic in the world, with an estimated 7.7 million people living with HIV (PLHIV) [1], and the highest incidence of new HIV infections globally [2]. In 2017, South Africa had an estimated 14.0% (95% CI 13.1% to 15.0%) population‐level HIV prevalence, with those aged 15 to 49 years having the highest estimated HIV prevalence (20.6%; 95% CI: 19.2% to 22.0%) [2]. While South Africa has made great strides in improving HIV testing services (HTS) (79.3% of females and 70.9% of males reported having ever been tested), by 2017, only 52.5% of females and 45.5% males aged 15 and older were aware of their HIV status, suggesting that South Africa had yet to reach its target of 90% of all people living with HIV knowing their HIV status. Although there is near universal coverage of HIV testing for pregnant women in antenatal clinics, access to and uptake of HTS by boys, men and non‐pregnant women remain sub‐optimal.

Both women and men face barriers to HTS, including navigating confidentiality concerns, distance to a testing facility, inconvenient operating hours and perceptions that facilities are mainly female spaces (i.e. provide maternal‐child health services only) [3, 4, 5]. Adolescent girls and young women (AGYW) are at increased risk for HIV infection and reduced testing uptake due to their unequal cultural, social and economic status [1, 6, 7, 8, 9, 10]. Furthermore, AGYW face clinic environments that perpetrate social and cultural judgement around their sexual behaviours, which impacts the access to and uptake of clinic‐based testing services [11]. Men, both young and old, have poorer HIV testing uptake which may emanate from the lack of comparable universal testing provided to women accessing antenatal care and family planning services, as well as masculinity norms around health and wellness [12, 13, 14]. This has resulted in a high rate of men not knowing their HIV status [2, 12, 15, 16]. Consequently, tailored HTS is needed.

Community‐based HIV counselling and testing (CBCT) has been shown to be acceptable to both men and women, and is an accepted platform to improve HTS coverage and uptake in South Africa [17]. Common CBCT modalities include systematic home‐based HCT (HIV Counselling and Testing), index testing and mobile HCT (near home and workplace). Interestingly, these modalities show different levels of efficacy depending on sex, age, risk groups and community environments, with little programmatic data to inform how to optimally implement and combine CBCT strategies to reach targeted population groups [18, 19]. Given the contextual realities on the ground, it remains unclear how CBCT platforms may be deployed to enhance, improve and target HIV testing and prevention services by sex and age groups. Towards this end, we present programmatic data from multimodal, at‐scale CBCT platforms implemented in 12 high HIV burden health districts in South Africa.

## 2 METHODS

We conducted an analysis of aggregate, HIV testing rates using programmatic data from a multimodal CBCT programme offered in South Africa.

### 2.1 CBCT programme design and delivery

**Figure 1:**
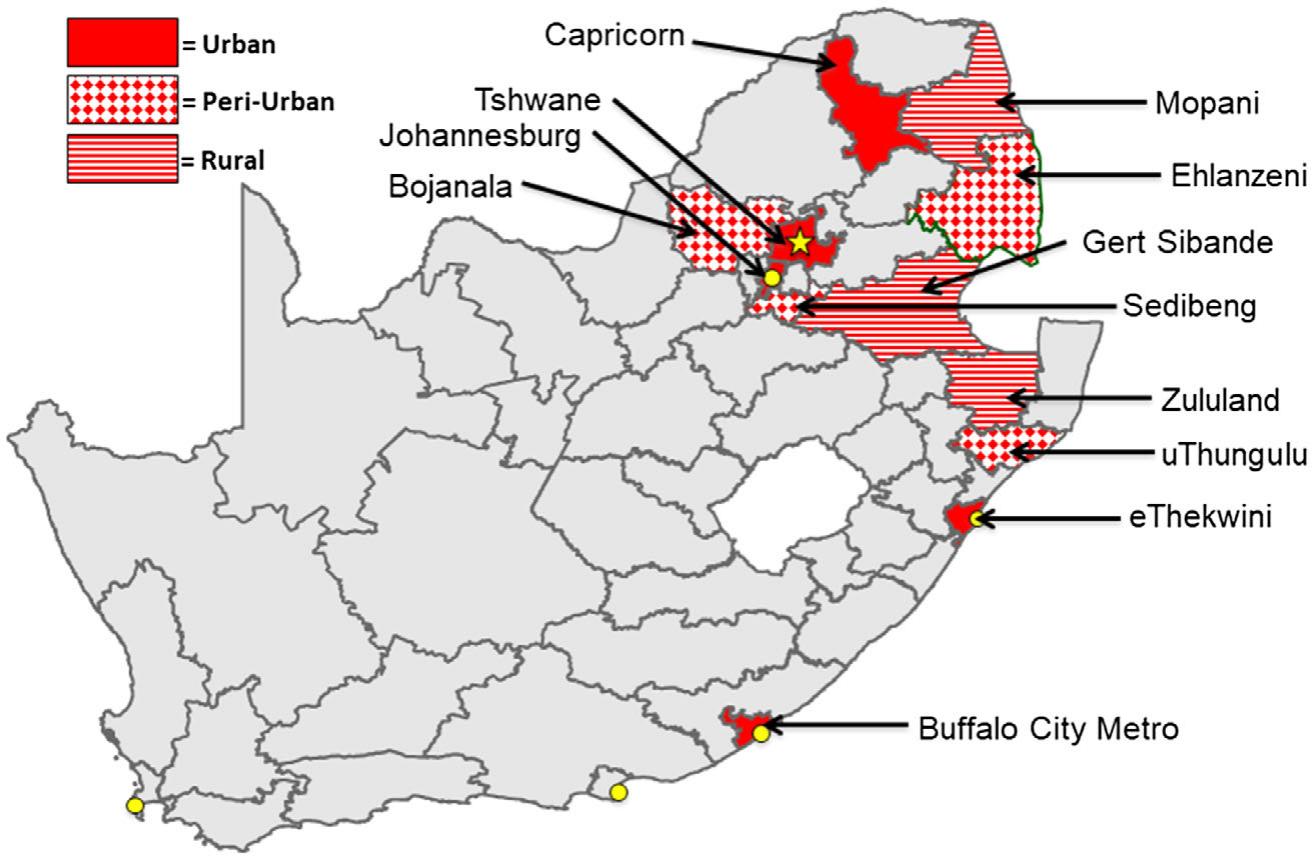
Map of South Africa’s 52 health districts. High HIV burden districts where multimodal community‐based HIV counselling and testing services were implemented are highlighted in red.

From January 2014 through November 2018, a comprehensive CBCT programme was implemented in 24 sub‐districts located in 12 “Focus for Impact” districts/local municipalities in South Africa (Figure 1; Table S1) [20]. District selection was driven by estimated HIV incidence and prevalence rates, number of PLHIV and estimated unmet anti‐retroviral therapy (ART) need at the district level as described [21]. As described in Table 1, the HTS modalities used included: systematic home‐based, index testing and differentiated targeting of mobile HTS including near‐home, workplace and twilight implementation. Additional descriptions of this CBCT programme can be found elsewhere [22, 23].

**Table 1:** Type and description of HIV testing service modalities implemented in 12 high burden districts in South Africa

HTS modalities	Description
Systematic home‐based testing	Systematic home‐based HTS entails household mapping at the community level and offering door‐to‐door HTS in the household to all households in the target community
Index testing	Index testing entails generating a list of sexual and household contacts of known HIV‐positive clients and offering HTS to these contacts. Index patients may include women living with HIV and engaged in antenatal care services, patients receiving antiretroviral therapy, tuberculosis patients and CBCT clients who test positive. The index testing model is encompassed within the systematic home‐based HTS model. Other than how individuals are identified for testing, the delivery of index testing HTS does not differ from that of systematic home‐based HTS
Workplace mobile unit testing	Workplace mobile HTS is offered at formal and informal work venues, as well as in industrial zones that are accessible to local and transient working populations. This approach targets areas and work venues with known high‐risk populations. This approach has been used to provide HTS to farmworkers, seasonal employees, migrant labourers, employees of the retail and automotive manufacturing sectors, mining industrial zones and transport workers (i.e. taxi drivers and truck drivers). HTS is offered during working hours, at or near the place of employment and with support and endorsement from labour unions and employers. Given that the majority of employees at such venues are men, this approach has traditionally been used to target men
Near‐home mobile unit testing	Near‐home mobile HTS entails testing providers driving a mobile testing unit to a pre‐determined location in a community and providing HTS from the mobile unit and/or auxiliary testing tents. The placement of a mobile unit is determined in consultation with local community leaders and health departments, and ensures that mobile units are accessible to individuals in the target populations and communities (e.g. youth centres, local commuter taxi ranks, major thoroughfares, special venues, on the outskirts of informal settlements). Mobile services can be offered during and after working hours, over the weekend and at special events
Twilight testing	Twilight HTS is designed to target clients (i.e. sex workers, MSM, people who inject drugs) in hotspots after 18:00. Twilight HTS implementation mirrors that of mobile HTS

Using aggregate, clinic‐level ART headcounts and HIV test positivity rates, heat‐maps were developed to identify and prioritize clinic catchment areas with the highest presumed HIV prevalence. Based on a sub‐district’s sociodemographic and economic profile, the clinic catchment area being targeted, and in consultation with local health departments, a combination of targeted and complementary CBCT testing teams, resources and modalities were concurrently implemented (i.e. combination implementation) to achieve the highest estimated HIV testing rates and positivity. Per South African National guidelines [24], any individual aged >12 years passively presenting for or actively offered an HIV test was tested; of note, all CBCT modalities, with the exception of home‐based testing, relied upon passive presentation for HTS at CBCT sites. The CBCT basic package of services was broken down into pre‐test, testing and post‐test services [21]. Post‐test counselling for those that received either a positive or negative HIV test result have been described previously [22, 23, 25].

### 2.2 Data collection

As previously described [23], paper‐based data collection tools were designed based on the South African Government (SAG) HCT registers and aligned to reporting requirements for the South African National Department of Health (NDoH) indicators, and donor reporting requirements [26]. SAG consent forms were used for all CBCT activities. Paper‐based data collection forms were completed by HIV testing counsellors, and entered into a Microsoft Access database by trained data capturers. The Foundation for Professional Development (FPD) monitoring and evaluation (M&E) support team performed data quality assurance, which included: regular data quality reviews, periodic site visits to review data collection and reporting processes, and structured data quality audits and data validation.

### 2.3 Data analysis

Aggregate, programmatic testing data collected from 1 October 2015 through 31 March 2017 were used in our analysis. South African National Census of 2011 data at sub‐district level were used as our denominators for testing rates, as mid‐year population estimates are only calculated to the provincial level [27]. Descriptive statistics were used to describe the data. Analysis outcomes included: HIV testing rates, defined as the number of tests performed per 100,000 population, and testing proportions by modality stratified by sex, age group and district were calculated; per‐100,000 statistics were used because the total number of people approached for testing was not captured. The “testing ratio” of women to men is calculated as: *# tests performed on women per 100,000 population/# tests performed on men per 100,000 population*. Districts were stratified into urban, peri‐urban and rural areas as described and defined by Statistics South Africa [28]. Where appropriate, 95% confidence intervals (95% CI) were calculated for proportions and testing rates to evaluate differences. South African National Census of 2011 standard population weights were used for direct age‐adjustments. Urban, peri‐urban and rural testing rates were calculated as age‐specific and overall age‐adjusted testing rates in order to compare within categories (i.e. urban vs. peri‐urban vs. rural). All analyses were carried out using STATA 13.0 (Stata Corporation, College Station TX, 2006).

### 2.4 Ethical considerations

Prior to receiving an HIV test, all individuals were counselled and consented per South African National HIV testing guidelines [29]. The Foundation for Professional Development Research Ethics Committee (FPD‐REC), Pretoria, South Africa, waived the need for ethics approval, as data used for this manuscript was aggregate, de‐identified programmatic data used for evaluation purposes. The FPD‐REC is accredited by the National Health Research Ethics Council of the South African Department of Health under registration number REC‐120208‐018.

## 3 RESULTS

### 3.1 HIV Testing rates per 100,000 population

**Figure 2:**
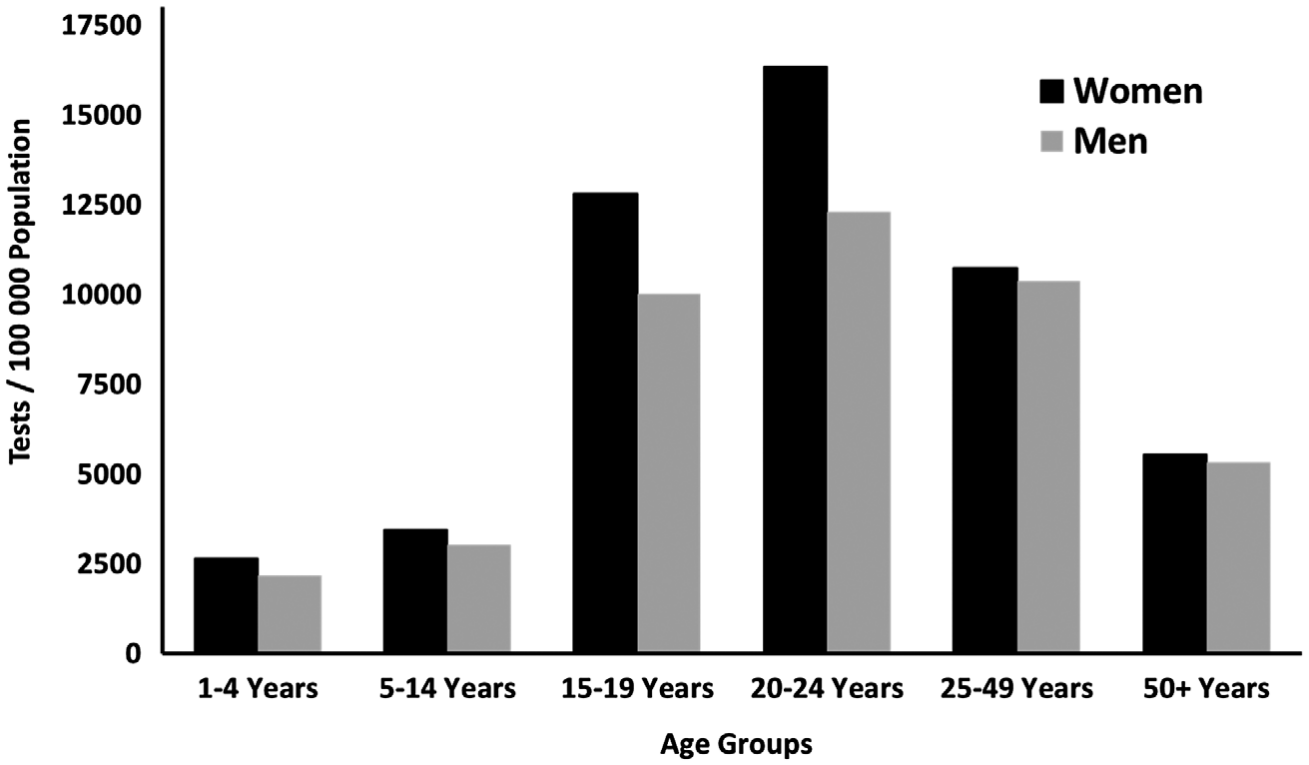
Aggregate HIV Test Performed per 100,000 population by Sex and Age Group from 12 High Burden Districts in South Africa Targeted for Multimodal CBCT Services, October 2015 to March 2017.

A total of 944,487 HIV tests were performed over the 1.5‐year period reported. The plurality of HIV tests were performed among clients aged 25 to 49 years (n = 455,484, 48.23%) with testing conducted among those aged one to four years (n = 27,579, 2.92%), five to fourteen years (n = 61,803, 6.54%), 15 to 19 years (n = 121,734, 12.89%), 20 to 24 years (n = 184,659, 19.55%) and 50+ years (n = 93,228, 9.87%) (Table 2). Of the total number of tests conducted, more females were tested compared to males (53.6% v. 46.4%), with an overall Female‐to‐Male (F:M) testing ratio of 1.11 (95% CI: 1.11 to 1.12; Table 2). Furthermore, HIV testing rates by females were consistently higher than that of males across all age groups (Figure 2).

**Table 2:** Aggregate total HIV test performed, tests per 100,000 population and female‐to‐male testing ratios in 12 high burden districts in South Africa by age group

	Total tests performed (% Total)	Overall testing rate Tests/100,000 (95% CI)	Female : male testing Ratio (95% CI)
All clients	944,487	8206.0 (8190.2 to 8221.9)	1.11 (1.11 to 1.12)
Age group (years)			
1 to 4	27,579 (2.92%)	2273.2 (2246.7 to 2299.9)	1.08 (1.06 to 1.11)
5 to 14	61,803 (6.54%)	3234.1 (3209.0 to 3259.2)	1.15 (1.13 to 1.16)
15 to 19	121,734 (12.89%)	11,432.8 (11372.4 to 11,493.4)	1.28 (1.27 to 1.29)
20 to 24	184,659 (19.55%)	14,303.8 (14,243.4 to 14,364.3)	1.33 (1.32 to 1.34)
25 to 49	455,484 (48.23%)	10,550.2 (10,521.2 to 10,579.2)	1.04 (1.03 to 1.04)
50+	93,228 (9.87%)	5444.4 (5410.5 to 5478.5)	1.04 (1.03 to 1.06)

The highest overall HIV testing rate per 100,000 people occurred among clients aged 20 to 24 years (14,303.8 per 100,000 people; 95% CI: 14,243.4 to 14,364.3), followed by those aged 15 to 19 years (11,432.8 per 100,000 people; 95% CI: 11,372.4 to 11,493.4) (Figure 2). More specifically, young women aged 15 to 19 and 20 to 24 had the highest testing rates of any age group of either sex (Figure 2), followed by young men aged 20 to 24 years (testing rate = 12,307.3 per 100,000 people; 95% CI: 12,227.5 to 12,387.4) (Figure 2).

When testing rates were stratified by sex, age band and district, young women aged 20 to 24 years continued to have the highest testing rates in all districts except Johannesburg and Zululand (Tables S2 and S3). In Johannesburg, young women aged 20 to 24 years had a testing rate similar to that of women aged 25 to 49 age, while in Zululand young men aged 20 to 24 years had a higher testing rate than young women (Tables S2 and S3). In Bojanala, Tshwane and uThungulu districts, men had higher testing rates compared to women (testing ratios of 0.84, 0.98 and 0.97 respectively; Table 3).

**Table 3:** District HIV testing rate per 100,000 population, October 2015 to March 2017

District	Overall testing rate	Female testing rate	Male testing rate	Female : male testing ratio
	Tests/100,000 population (95% CI)	Tests /100,000 population (95% CI)	Tests /100,000 population (95% CI)	Testing ratio (95% CI)
Bojanala	5984.5 (5932.4 to 6037.0)	5418.1 (5345.3 to 5491.5)	6480.5 (6406.5 to 6555.2)	0.84 (0.82 to 0.85)
Buffalo City	9422.42(9356.6 to 9488.5)	10,478.0 (10,382.8 to 10,573.7)	8254.8 (8164.9 to 8345.3)	1.27 (1.25 to 1.29)
Capricorn	6798.3 (6743.0 to 6854.0)	6946.6 (6869.5 to 7024.4)	6634.8 (6555.4 to 6714.7)	1.05 (1.03 to 1.06)
Ehlanzeni	9007.7 (8938.5 to 9077.2)	10,005.3 (9903.9 to 10,107.4)	7966.0 (7872.5 to 8060.2)	1.26 (1.24 to 1.28)
eThekwini	2880.4 (2862.8 to 2898.1)	2879.7 (2855.0 to 2904.5)	2881.2 (2855.9 to 2906.6)	1.00 (0.99 to 1.01)
Gert Sibande	24,085.1 (23,959.4 to 24,211.2)	25,954.2 (25,770.1 to 26,139.0)	22,288.2 (22,116.9 to 22,460.2)	1.16 (1.15 to 1.18)
Johannesburg	10,371.7 (10,297.0 to 10,446.8)	11,999.8 (11,884.1 to 12,116.3)	8910.9 (8814.8 to 9007.7)	1.35 (1.33 to 1.37)
Mopani	11,417.8 (11,339.7 to 11,496.2)	13,488.0 (13,372.9 to 1360.7)	9074.8 (8971.9 to 9178.5)	1.49 (1.47 to 1.51)
Sedibeng	5141.2 (5093.4 to 5189.3)	5190.6 (5123.1 to 5258.7)	5090.6 (5023.1 to 5158.9)	1.02 (1.00 to 1.04)
Tshwane	6666.3 (6625.3 to 6707.5)	6604.5 (6547.0 to 6662.4)	6728.9 (6670.5 to 6787.7)	0.98 (0.97 to 0.99)
uThungulu	17,975.7 (17,874.2 to 18,077.7)	17,712.7 (17,573.1 to 17,852.9)	18,264.3 (18,116.4 to 18,413.0)	0.97 (0.96 to 0.98)
Zululand	34,253.3 (34,087.7 to 34,419.20)	35,315.2 (35,088.5 to 35,542.5)	33,000.9 (32,758.7 to 33,243.8)	1.07 (1.06 to 1.08)

The overall age‐adjusted testing rate was highest among rural areas (21,230.9 per 100,000 people) followed by peri‐urban (8,315.1 per 100,000 people) and then urban setting (5198.5 per 100,000 people) (Table 4). This pattern continued with the overall age‐specific rates by sex. Notably, the age group 20 to 24 years had the overall highest age‐specific testing rates across the geographical areas. When disaggregated by sex, females had higher age‐adjusted testing rates than the males in urban and rural areas. In peri‐urban areas, the age‐adjusted testing rates were similar for females and males (Table 4). In urban areas, men aged 50+ year had a notably higher age‐specific testing rate compared to females aged 50+ years (Table 4). In peri‐urban districts, males aged 20 to 24 years (15,123.4 per 100,000 people; 95% CI: 14,945.0 to 15,303.2) and females age 15 to 19 years (15,133.3 per 100,000 people; 95% CI: 14,940.7 to 15,327.4) had the highest testing rates (Table 4).

**Table 4:** HIV testing rates per 100,000 persons by district‐level geographical classification stratified by sex and age group

Class	1 to 4 years (95% CI)	5 to 14 years (95% CI)	15 to 19 years (95% CI)	20 to 24 years (95% CI)	25 to 49 years (95% CI)	50+ years (95% CI)	Age‐adjusted testing rate per 100,000 persons^a^
Overall							
Urban	1366.3(1339.4 to 1393.5)	1571.9(1548.8 to 1595.3)	6583.9(6522.6 to 6645.6)	9607.6(9543.7 to 9671.8)	7546.7(7515.4 to 7578.0)	3009.0(2976.5 to 3041.8)	5198.5(5162.6 to 5234.6)
Peri‐Urban	2967.7(2907.6 to 3028.8)	4525.2(4466.5 to 4584.5)	10,812.7(10,703.1 to 10,923.1)	13,466.5(13,353.1 to 13,581.6)	10,603.4(10,544.5 to 10,662.6)	6290.8(6217.4 to 6364.8)	8315.1(8243.1 to 8387.8)
Rural	4686.2(4586.5 to 4787.5)	7158.4(7064.1 to 7253.5)	28,835.9(28,606.9 to 29,065.7)	37,925.7(37,676.6 to 38,175.3)	28,451.8(28,319.7 to 28,584.2)	16,991.7(16,825.0 to 17,159.4)	21,230.9(21,082.0 to 21,380.6)
Female							
Urban	1447.8(1408.7 to 1487.8)	1706.7(1672.5 to 1741.5)	7608.5(7516.5 to 7701.2)	11,279.0(11,181.7 to 11,376.9)	7607.8(7563.4 to 7652.4)	2760.4(2719.1 to 2802.2)	5487.4(5386.2 to 5489.4)
Peri‐Urban	3285.9(3196.4 to 3377.2)	5204.2(5115.1 to 5294.4)	15,133.3(14,940.7 to 15,327.4)	12,883.4(12,742.3 to 13,025.5)	11,488.5(11,400.8 to 11,576.6)	6973.7(6869.2 to 7079.4)	9207.4(9101.0 to 9314.8)
Rural	4888.6(4745.3 to 5035.1)	7832.5(7693.1 to 7973.7)	33,239.2(32,902.3 to 33,577.5)	43,381.6(43,020.2 to 43,743.5)	29,213.8(29,030.9 to 29,397.1)	18,732.5(18,505.7 to 18,960.8)	22,855.1(22,643.7 to 23,067.7)
Male							
Urban	1286.2(1249.6 to 1323.6)	1444.1(1412.9 to 1475.8)	5573.8(5493.1 to 5655.3)	7998.5(7915.6 to 8082.0)	7485.8(7441.8 to 7529.9)	3338.4(3286.3 to 3391.1)	4929.5(4879.9 to 4979.6)
Peri‐Urban	3209.7(3121.8 to 3299.4)	4850.6(4765.2 to 4937.0)	13,240.3(13,056.0 to 13,426.3)	15,123.4(14,945.0 to 15,303.2)	11,723.8(11,638.1 to 11,810.0)	6609.4(6498.6 to 6721.5)	9262.2(9153.1 to 9372.3)
Rural	4482.2(4344.1 to 4623.4)	6495.7(6369.0 to 6624.1)	24,434.3(24,127.4 to 24,743.2)	32,547.8(32,209.0 to 32,888.0)	27,591.1(27,400.1 to 27,782.6)	14,560.8(14,318.7 to 14,805.4)	19,386.8(19,179.7 to 19,595.5)

^a^ South African National Census of 2011 standard population weights were used for direct age‐adjustment.

### 3.2 HIV testing proportions stratified by modality

Home‐based testing accounted for the majority (n = 578,548; 61.3%) of HIV tests performed, followed by near‐home mobile unit testing (n = 285,572; 30.2%). Workplace mobile unit testing (n = 44,281; 4.7%), index testing (n = 27,794; 2.9%) and twilight testing (n = 8,292; 0.9%) accounted for a small proportion of the total tests performed (Table 5). A higher proportion of females than males were tested via home‐based HTS (56.4% [95% CI: 56.2 to 56.5] vs. 43.6% [95% CI: 43.5 to 43.8]); similar proportion were observed for index testing (56.7% [95% CI: 56.1 to 57.3] vs. 43.3 [95% CI: 42.7 to 43.9]). Among those tested, more males compared to females accessed HTS via workplace (57.4% [95% CI: 56.9 to 57.9] vs. 42.6 [95% CI: 42.1 to 43.1]) and twilight (58.1% [95% CI: 57.0 to 59.2] vs. 41.9 [95% CI: 40.8 to 43.0]) testing modalities. For near‐home mobile unit testing, there was no difference in testing proportions between men and women.

**Table 5:** HIV testing proportions by HTS modality by sex and age group

Characteristic	Home based %; 95 CI% (n)	Index testing %; 95 CI% (n)	Mobile near home %; 95 CI% (n)	Mobile twilight %; 95 CI% (n)	Mobile workplace %; 95 CI% (n)
All clients (row %)	61.3; 61.2 to 61.4 (n = 578,548)	2.9; 2.9 to 3.0 (n = 27,794)	30.2; 30.1 to 30.3 (n = 285,572)	0.9; 0.9 to 0.9 (n = 8292)	4.7; 4.6 to 4.7 (n = 44,281)
Sex (column %)					
Female	56.4; 56.2 to 56.5 (n = 326,020)	56.7; 56.1 to 57.3 (n = 15,750)	49.6; 49.4 to 49.8 (n = 141,729)	41.9; 40.8 to 43.0 (n = 3474)	42.6; 42.1 to 43.1 (n = 18,867)
Male	43.6; 43.5 to 43.8 (n = 252,528)	43.3; 42.7 to 43.9 (n = 12,044)	50.4; 50.2 to 50.6 (n = 143,843)	58.1; 57.0 to 59.2 (n = 4818)	57.4; 56.9 to 57.9 (n = 25,414)
Age Group (column %)					
1 to 4 years	3.7; 3.6 to 3.7 (n = 21,338)	8.8; 8.4 to 9.1 (n = 2438)	1.3; 1.3 to 1.4 (n = 3741)	0.4; 0.3 to 0.5 (n = 32)	0.1; 0.0 to 0.1 (n = 30)
5 to 14 years	8.0; 7.9 to 8.0 (n = 45,999)	19.5; 19.0 to 19.9 (n = 5413)	3.6; 3.5 to 3.7 (n = 10,246)	1.1; 0.9 to 1.3 (n = 89)	0.1; 0.1 to 0.2 (n = 56)
15 to 19 years	13.7; 13.6 to 13.7 (n = 79,004)	10.8; 10.4 to 11.2 (n = 3000)	13.1; 13.0 to 13.3 (n = 37,490)	8.9; 8.2 to 9.5 (n = 734)	3.4; 3.2 to 3.6 (n = 1506)
20 to 24 years	18.2; 18.1 to 18.3 (n = 105,141)	13.2; 12.8 to 13.6 (n = 3660)	23.6; 23.4 to 23.7 (n = 67,364)	20.7; 19.8 to 21.6 (n = 1717)	15.3; 15.0 to 15.6 (n = 6777)
25 to 49 years	45.9; 45.8 to 6.0 (n = 265,503)	40.0; 39.4 to 40.6 (n = 11,113)	49.9; 49.7 to 50.1 (n = 142,520)	62.0; 60.9 to 63.0 (n = 5137)	70.5; 70.1 to 70.9 (n = 31,211)
50+ years	10.6; 10.5 to 10.7 (n = 61,563)	7.8; 7.5 to 8.1 (n = 2170)	8.5; 8.4 to 8.6 (n = 24,211)	7.0; 6.5 to 7.6 (n = 583)	10.6; 10.3 to 10.9 (n = 4701)

The highest proportion of those tested for each modality were clients aged 25 to 49 years, ranging from 40.0% for those accessing HTS via index testing to 70.5% for workplace testing (Table 5). For individuals under the age of 15 years, index testing was proportionately the most utilized modality among this age group (Table 5). Home‐based testing services were proportionately the most utilized modality by those aged 15 to 19 years (13.8%; 95% CI: 13.7 to 13.9). Among those aged 20 to 24 years, near‐home mobile testing was proportionately the most utilized modality accessed by this age group (23.6%; 95% CI: 23.4 to 23.7) (Table 5). Among those aged 25 to 49 years, workplace mobile testing was proportionately the most utilized modality among this age group (70.5%; 95% CI: 70.1 to 70.5). Finally, among those aged 50+ years, home‐based and workplace testing were proportionately equal and the most utilized modalities at providing community‐based HTS to this age group (10.6%; 95% CI: 10.5 to 10.7 and 10.6%; 95% CI: 10.3 to 10.9 respectively).

## 4 DISCUSSION

Improving HIV testing rates is key to achieving the second and third targets of the UNAIDS 90‐90‐90 and future pending 95‐95‐95 targets. However, traditional clinic‐based approaches to HTS will be insufficient for South Africa to reach its 90‐90‐90 targets and future pending 95‐95‐95 targets. CBCT programmes using multiple HTS modalities have the potential to overcome barriers associated with facility‐based testing and reduce testing coverage gaps [3, 18, 30, 31, 32, 33, 34]. Our CBCT data show that aggregate HIV testing rates for females was slightly higher than that of the males, mirroring what has been repeatedly documented across the SSA region – that men are less likely to be tested for HIV or seek and initiate antiretroviral therapy (ART) [13, 35, 36, 37, 38]. However, previous clinic‐based intervention studies have reported HIV testing ratios ranging from 1.7 to 2.2 [29, 35, 39, 40]. Our implemented CBCT programme resulted in an overall F:M testing ratio of 1.1. Moreover, we found a significantly higher testing rate by males in three of our 12 priority districts due to accessing of mobile, near‐home HTS by men. Our findings are similar to previous studies that showed mobile CBCT platforms located in convenient and accessible sites were effective at reaching men for HIV testing, with one study demonstrating higher rates of HTS uptake for men compared to women [16, 41, 42, 43].

Like men, young people (men and women) in African settings are reluctant to use health facilities [16, 44]. Our programmatic data revealed that adolescents and young adults aged 15 to 24 years had among the highest testing rates, with adolescent girls and young women having the highest overall testing rates. Specifically, young women aged 20 to 24 had the highest testing rates overall, a phenomenon that occurred in 11 of 12 districts (only women aged 25 to 49 years in Johannesburg had a higher, but insignificant, testing rate), followed by adolescent girls aged 15 to 19 years, which had the second‐highest testing rates in 7 of 12 districts. Among men, the highest overall testing rate was among those aged 20 to 24 years. These findings indicate that our targeted, multimodality CBCT programme was well positioned within communities and effective for delivering HTS to young women and men [16, 45]. This is consistent with other findings that CBCT acceptability by youth is dependent on its delivery in youth spaces in African settings [16, 46].

## 5 LIMITATIONS

Given the programmatic nature of our work, there are a number of limitations regarding the interpretation or generalization of our findings. Due to differential allocation of CBCT resources to meet donor targets in each district, we were unable to compare testing rates and proportions across testing modalities and districts. Given that communities were not presented with all possible modalities at the same time, we are limited in our ability to speak to the unique profiles of people that would prefer one CBCT modality over the other, or rigorously assess which modalities may have worked best given the sociodemographic and socioeconomic profiles of a district. Our use of aggregated testing data, not unique clients tested, likely results in some overestimation of the testing rates per 100,000 population. Moreover, given that mid‐year population estimates are only available at a provincial level, not at the district or sub‐district level, we are limited in our ability to adjust for population changes (i.e. growth or migration rates) since 2011. Moreover, given the lack of information on HIV prevalence estimates at sub‐district level or the number of first‐time or repeat testers, the testing rates may be slightly overestimated. Finally, no data were available for a cost‐effectiveness analysis to fully understand the economic value of each modality.

## 6 CONCLUSIONS

The success of this CBCT programme in reaching such high testing rates in such a short amount of time is likely due in part to the significant diversity and intensity of community‐based testing modalities implemented. Given the costs associated with this programme, replicating its success may be difficult. However, given the consultations with community and health leaders in determining which modalities may be best to utilize in their communities, significant improvements in the accessibility, and thus uptake of testing, can be replicated elsewhere. Our multimodal CBCT programme was effective at reaching adolescent girls and young women, and decreasing the testing gap between men and women. Given the anticipated at‐scale roll out of pre‐exposure prophylaxis (PrEP) in South Africa, the South African National Department of Health, and its supporting donors, should consider leveraging community‐based delivery platforms for PrEP services to maximize young women’s access and uptake of HIV prevention services. Although we found that some testing modalities seemed to be more readily acceptable and accessible to men, CBCT programmes should be further tailored to optimize testing uptake by males to address their HIV prevention and treatment barriers. Ultimately, implementing multiple CBCT modalities will help men, women and adolescents navigate specific HIV testing barriers and ensure reach and uptake so to meet the 90‐90‐90 targets in South Africa.

## COMPETING INTEREST

The authors have declared no conflict of interest.

## AUTHORS’ CONTRIBUTIONS

S. J. and N. N. secured funding. T. F, J. S., G. G., S. J. and N. N. implemented the programme. J. S. and G. G. managed and curated databases. A. M. M. developed data analysis plan. A. M. M., S. J., N. N. and T. F. performed analysis. A. M. M., L. G. B. and S. J. interpreted and contextualized findings. A. M. M, J. D., D. B. and R. P. wrote the paper. All authors have read and approved the final manuscript.

## Supporting information


**Table S1.** High HIV burden sub‐districts where multimodal community‐based HIV counselling and testing services were implemented, October 2015 to March 2017
