# Supplementary material for: Outcomes from a multimodal, at‐scale community‐based HIV counselling and testing programme in twelve high HIV burden districts in South Africa
Source: J Int AIDS Soc. 2021 Mar 11;24(3):e25678. doi: 10.1002/jia2.25678 (PMC7952633; doi:10.1002/jia2.25678)
Supplement: Supplementary file 1 — Table S1. High HIV burden sub‐districts where multimodal community‐based HIV counselling and testing services were implemented, October 2015 to March 2017 [file JIA2-24-e25678-s001.docx]

**SUPPLEMENTAL TABLES**

| **Supplemental Table 1. High HIV burden sub-districts where multimodal community-based HIV counselling and testing services were implemented, October 2015 – March 2017** | | |
| --- | --- | --- |
| **PROVINCE** | **DISTRICT** | **SUB-DISTRICT** |
| Eastern Cape | Buffalo City | Buffalo City Metropolitan |
| Gauteng | City of Johannesburg | Johannesburg Region A |
|  |  | Johannesburg Region D |
|  |  | Johannesburg Region E |
|  |  | Johannesburg Region G |
|  | City of Tshwane | Tshwane Sub-district 1 |
|  |  | Tshwane Sub-district 3 |
|  |  | Tshwane Sub-district 6 |
|  | Sedibeng | Emfuleni |
|  |  | Midvaal |
| KwaZulu Natal | eThekwini | eThekwini Metropolitan |
|  | uThungulu | uMhlathuze |
|  |  | uMlalazi |
|  | Zululand | Ulundi |
|  |  | uPhongolo |
| Limpopo | Capricorn | Polokwane |
|  | Mopani | Tzaneen |
|  |  | Giyani |
| Mpumalanga | Ehlanzeni | Mbombela |
|  |  | Nkomazi |
|  | Gert Sibande | Govan Mbeki |
|  |  | Msukalingwa |
|  |  | Lekwa |
| North West | Bojanala | Rustenburg |

| **Supplemental Table 2. HIV tests performed per 100,000 population for females by age group and district, October 2015 – March 2017** | | | | | | |  |
| --- | --- | --- | --- | --- | --- | --- | --- |
| **District** | **1 – 4 years** | **5 – 14 years** | **15 – 19 years** | **20 – 24 years** | **25 – 49 years** | **50+ years** | |
|  | **tests /100,000 (95% CI)** | **tests /100,000 (95% CI)** | **tests /100,000 (95% CI)** | **tests /100,000 (95% CI)** | **tests /100,000 (95% CI)** | **tests /100,000 (95% CI)** | |
| Bojanala | 2197.7 (2060.0-2342.1) | 2046.6 (1933.2-2164.9) | 6990.7 (6705.7-7283.9) | 10521.2 (10215.4-10833.1) | 7156.91 (7022.5-7293.1) | 2875.6 (2744.0-3011.7) | |
| Buffalo City | 965.7 (870.1-1068.9) | 2737.4 (2608.1-2871.3) | 17618.4 (17218.7-18023.5) | 21553.2 (21145.5-21965.2) | 14851.0 (14668.1-15035.4) | 4457.5 (4315.9-4602.3) | |
| Capricorn | 2040.3(1913.3-2173.4) | 1870.6 (1777.1-1967.6) | 8995.1 (8730.8-9264.7) | 17469.8 (17110.5-17833.6) | 9472.0 (9315.9-9629.8) | 3270.0 (3138.3-3405.6) | |
| Ehlanzeni | 683.4 (601.1-773.8) | 1429.4 (1335.9-1527.7) | 9271.0 (8964.6-9584.4) | 18551.2 (18154.1-18953.3) | 17165.3 (16953.5-17378.7) | 3524.6 (3359.9-3695.1) | |
| eThekwini | 767.1 (725.2-810.7) | 954.6 (918.0-992.2) | 3650.5 (3559.7-3742.8) | 5868.8 (5767.4-5971.4) | 3645.9 (3600.8-3691.5) | 1616.3 (1571.9-1661.7) | |
| Gert Sibande | 882.9 (767.2-1011.1) | 5015.8 (4799.9-5238.6) | 44637.4 (43953.3-45323.1) | 61738.4 (61097.5-62376.2) | 34278.3 (33947.5-34610.3) | 12795.0 (12440.7-13155.7) | |
| Johannesburg | 3287.2 (3104.0-3478.1) | 4800.0 (4585.7-5021.4) | 11595.9 (11145.8-12057.6) | 16002.1 (15642.6-16366.6) | 16225.6 (16035.5-16417.0) | 5051.9 (4788.9-5324.9) | |
| Mopani | 1719.8 (1594.2-1852.6) | 3420.1 (3279.3-3565.2) | 17642.1 (17242.1-18047.6) | 24377.0 (23913.5-24844.9) | 19620.8 (19389.5-19853.7) | 11752.2 (11483.1-12025.4) | |
| Sedibeng | 1195.4 (1091.1-1306.9) | 1815.0 (1712.2-1922.3) | 9683.1 (9381.7-9990.9) | 10328.1 (10043.3-10618.3) | 5569.9 (5455.4-5686.2) | 4249.5 (4108.5-4394.0) | |
| Tshwane | 1963.3 (1862.3-2068.3) | 1782.4 (1702.1-1865.4) | 10114.01 (9868.0-10364.2) | 14328.1 (14083.8-14575.1) | 7858.4 (7759.7-7957.9) | 3742.8 (3636.1-3851.7) | |
| uThungulu | 8797.7 (8490.9-9112.0) | 13995.8 (13708.8-14286.5) | 28522.0 (28026.0-29021.9) | 29761.5 (29265.6-30261.0) | 14533.6 (14307.3-14762.1) | 18995.8 (18619.6-19376.4) | |
| Zululand | 14615.8 (14157.5-15083.2) | 17393.0 (17024.1-17766.6) | 48594.6 (47900.9-49288.8) | 55844.5 (55098.3-56588.8) | 44174.3 (43717.8-44631.5) | 42020.3 (41407.2-42635.4) | |

| **Supplemental Table 3. HIV tests performed per 100,000 population for males by age group and district, October 2015 – March 2017** | | | | | | |
| --- | --- | --- | --- | --- | --- | --- |
| **District** | **1 – 4 years** | **5 – 14 years** | **15 – 19 years** | **20 – 24 years** | **25 – 49 years** | **50+ years** |
|  | **tests /100,000 (95% CI)** | **tests /100,000 (95% CI)** | **tests /100,000 (95% CI)** | **tests /100,000 (95% CI)** | **tests /100,000 (95% CI)** | **tests /100,000 (95% CI)** |
| Bojanala | 2230.0 (2092.7-2373.8) | 2421.2 (2299.3-2547.7) | 6789.6 (6514.7-7072.6) | 9132.3 (8863.9-9406.2) | 8750.3 (8621.3-8880.7) | 4589.5 (4424.9-4758.4) |
| Buffalo City | 865.3 (776.13-961.9) | 2023.9 (1914.5-2137.8) | 9911.0 (9592.3-10236.6) | 13505.4 (13161.7-13854.7) | 13172.4 (12988.1-13358.2) | 4599.4 (4429.5-4773.9) |
| Capricorn | 1620.1 (1507.8-1738.4) | 1592.1 (1507.0-1680.7) | 5550.3 (5339.6-5766.9) | 11796.1 (11495.0-12102.3) | 11530.6 ( 11346.0-11717.1) | 4596.5 (4406.7-4792.1) |
| Ehlanzeni | 552.7 (479.8-633.5) | 1269.7 (1182.2-1361.8) | 6375.8 (6113.1-6646.3) | 12355.1 (12019.6-12696.7) | 13968.4 (13769.7-14169.0) | 4645.9 (4434.8-4864.1) |
| eThekwini | 666.3 (627.7-706.7) | 754.1 (721.9-787.4) | 3165.4 (3079.1-3253.4) | 4746.0 (4655.0-4838.2) | 4078.8 (4031.1-4126.9) | 1650.1 (1597.7-1703.8) |
| Gert Sibande | 840.7 (727.8-966.1) | 3656.7 (3473.2-3847.1) | 32359.3 (31717.8-33005.7) | 43016.0 (42405.5-43628.3) | 31974.5 (31665.6-32284.6) | 10908.3 (10559.6-11264.4) |
| Johannesburg | 2882.5 (2712.5-3060.2) | 4494.3 (4288.6-4706.9) | 13681.6 ( 13190.8-14183.7) | 10284.3 (9989.1-10585.1) | 10805.9 (10661.3-10951.7) | 5492.0 (5222.3-5771.3) |
| Mopani | 1523.9 (1405.7-1649.1) | 2689.5 (2565.6-2817.7) | 10770.8 (10446.1-11102.2) | 13730.9 (13364.1-14104.1) | 14718.9 (14487.6-14952.5) | 8762.4 (8474.2-9057.2) |
| Sedibeng | 1168.8 (1065.4-1279.5) | 1547.6 (1453.3-1646.3) | 9397.8 (9093.9-9708.5) | 8704.4 (8444.7-8969.5) | 5878.4 (5762.2-5996.2) | 4271.5 (4116.9-4430.2) |
| Tshwane | 1926.5 (1827.0-2029.9) | 1668.1 (1590.8-1748.2) | 7041.8 (6830.9-7257.3) | 10638.1 (10425.2-10853.8) | 9045.1 (8941.7-9149.2) | 5241.2 (5103.6-5381.5) |
| uThungulu | 8817.5 (8511.6-9130.7) | 13056.7 (12781.2-13336.0) | 26065.9 (25570.7-26565.6) | 28972.5 (28453.3-29496.0) | 19875.4 (19605.6-20147.3) | 15506.5 (15088.7-15931.2) |
| Zululand | 13762.5 (13308.5-14226.2) | 15193.7 (14846.9-15545.5) | 39918.4 (39240.1-40599.5) | 57543.0 (56736.4-58346.5) | 50902.6 (50362.2-51442.7) | 37083.4 (36291.2-37880.8) |
